# APKASS 2024 consensus statement on anterior cruciate ligament reconstruction, Part II: Management of anterior cruciate ligament revision in adults

**DOI:** 10.1016/j.asmart.2026.06.001

**Published:** 2026-08-13

**Authors:** Mingde Cao, Yuichi Hoshino, Gilbert Moatshe, Joon Ho Wang, Pakapon Issaragrisil, Stefan Mogos, Nadhaporn Saengpetch, Chanakarn Phornphutkul, Jin Goo Kim, Ryosuke Kuroda, Nattha Kulkamthorn, Hideyuki Koga, Shuhei Otsuki, Ryuichiro Akagi, Sang-Hak Lee, Karl Eriksson, Kyoung Ho Yoon, Bancha Chernchujit, Ekavit Keyurapan, Vasileios Raoulis, Rainer Siebold, Jonathan Patrick Ng, Patrick Shu-Hang Yung, Michael Tim-Yun Ong

**Affiliations:** a Department of Orthopaedics & Traumatology, Faculty of Medicine, the Chinese University of Hong Kong, Hong Kong Special Administrative Region of China; b Department of Orthopaedic Surgery, Kobe University Graduate School of Medicine, Kobe City, Japan; c Oslo Sports Trauma Research Center, Norwegian School of Sport Sciences, Oslo, Norway; d Department of Orthopaedic Surgery, Samsung Medical Center, Sungkyunkwan University School of Medicine, Seoul, Republic of Korea; e Bangkok Academy of Sports and Exercise Medicine (BASEM), FIFA Medical Center of Excellence-FIFA, Bangkok Hospital Headquarters, Bangkok, Thailand; f Nord Hospital, Bucharest, Romania; g Department of Orthopaedics, Faculty of Medicine Ramathibodi Hospital, Mahidol University, Bangkok, Thailand; h Department of Orthopaedics, Faculty of Medicine, Chiangmai University, Chiangmai, Thailand; i Department of Orthopedic Surgery and Sports Center, Myong-Ji Hospital, Seoul, Republic of Korea; j Department of Orthopaedics, Phramongkutklao Hospital and College of Medicine, Bangkok, Thailand; k Department of Joint Surgery and Sports Medicine, Graduate School of Medical and Dental Sciences, Institute of Science Tokyo, Tokyo, Japan; l Department of Orthopedic Surgery, Osaka Medical and Pharmaceutical University, Takatsuki, Japan; m Knee Surgery and Sports Medicine Center, Oyumino Central Hospital, Chiba, Chiba, Japan; n Department of Orthopedic Surgery, Center for Joint Diseases and Rheumatism, Kyung Hee University Hospital, Seoul, Republic of Korea; o Department of Orthopaedics, Stockholm South Hospital, Karolinska Institutet, Stockholm, Sweden; p Department of Orthopedic Surgery, Kyung Hee University Hospital, Seoul, Republic of Korea; q Department of Orthopaedics, Faculty of Medicine, Thammasat University, Bangkok, Thailand; r Faculty of Medicine, Siriraj Hospital, Mahidol University, Bangkok, Thailand; s Department of Sports Medicine, University Hospital of Larissa, Larissa, Greece; t International Center for Orthopedics, ATOS Clinic, Heidelberg, Germany

**Keywords:** Consensus statement, Diagnosis, Graft selection, Lateral extra-articular tenodesis, Revision ACL reconstruction, Surgical indication

## Abstract

**Background:**

Revision anterior cruciate ligament reconstruction (ACLR) is a complex procedure with varying diagnostic, surgical, and rehabilitation approaches. This study aimed to develop a consensus among surgeons from APKASS on key aspects of revision ACLR to improve clinical outcomes.

**Methods:**

A total of 23 expert surgeons from eight member countries participated in the survey. Topics included diagnostics, surgical indications, techniques, graft selection, rehabilitation protocols, and return-to-play (RTP) considerations for revision ACLR. Responses were analyzed in comparison with the current literature to identify areas of agreement and divergence.

**Results:**

A consensus was reached on the importance of clinical and imaging criteria, including the pivot shift test, KT-2000 measurements, and MRI for identifying ACL failure. CT was preferred for evaluating tunnel placement. Poor tunnel placement was identified as the leading cause of ACL failure. One-stage revision surgeries were favoured unless significant tunnel enlargement required a two-stage approach. Bone-patellar tendon-bone (BTB) autografts were the preferred graft choice, while quadriceps tendon (QT) autografts emerged as a viable alternative. Revision ACLR was deemed critical for active patients engaged in high-impact sports, while older age and established osteoarthritis were considered relative contraindications. Additional procedures, such as lateral extra-articular tenodesis (LET), were selectively indicated based on individual patient factors. Conservative, patient-specific rehabilitation protocols were recommended, with an emphasis on addressing psychological factors. RTP rates varied widely, highlighting the importance of tailored recovery plans to optimize outcomes.

**Conclusion:**

This consensus emphasizes the importance of individualized and evidence-based approaches in revision ACLR, from diagnostics and surgical techniques to rehabilitation and RTP. Standardized criteria and further research are essential to refine strategies and improve outcomes in this patient population.

**Level of evidence:**

V (Expert opinion).

## 1 Introduction

Revision anterior cruciate ligament reconstruction (ACLR) is growing in importance as an essential procedure, driven by the increasing number of primary ACLR worldwide.^1^ A systematic review summarized the primary ACLR performed between 1969 and 2018, the pooled revision rate ranged from 2.76% to 3.56%.^2^ Although the overall revision rate is relatively low, the number of revision cases is still sizable. Furthermore, clinical outcomes for patients undergoing revision ACLR tend to be less favourable compared to primary ACLR in terms of IKDC score and Lysholm score,^3^^,^^4^ with re-revision rates documented 3∼4 times higher than that of primary reconstruction.^5^ In light of these challenges, the development and implementation of more standardized treatment paradigms may facilitate improved clinical outcomes for patients.

Despite advancements in surgical techniques, numerous controversies and challenges persist in revision ACLR, including the optimal diagnostic approach, appropriate preoperative planning, indications for one-stage versus two-stage procedures, graft selection, the role of lateral augmentation, and even the rehabilitation protocols. The absence of consensus and standardized evidence in the existing literature on these topics often compels surgeons to rely on their clinical expertise and institutional protocols to guide decision-making.

Recognizing these gaps, the APKASS consensus paper builds upon prior discussions^6^, ^7^, ^8^, ^9^, while expanding the scope to include diverse perspectives—particularly from experts across Asia-Pacific countries. This region's unique patient demographics and surgical expertise provide valuable insights into the nuances of revision ACLR surgery, enabling a broader and more inclusive understanding of best practices.

Given the limited evidence-based recommendations available for revision ACLR, this study aims to address the most debated aspects of revision ACLR. By leveraging the collective expertise of knee surgeons across the Asia-Pacific region, the resulting recommendations aim to provide practical guidance for orthopaedic surgeons worldwide and reflect the distinctive challenges and considerations faced in Asian clinical settings.

## 2 Materials and methods

**Fig. 1:**
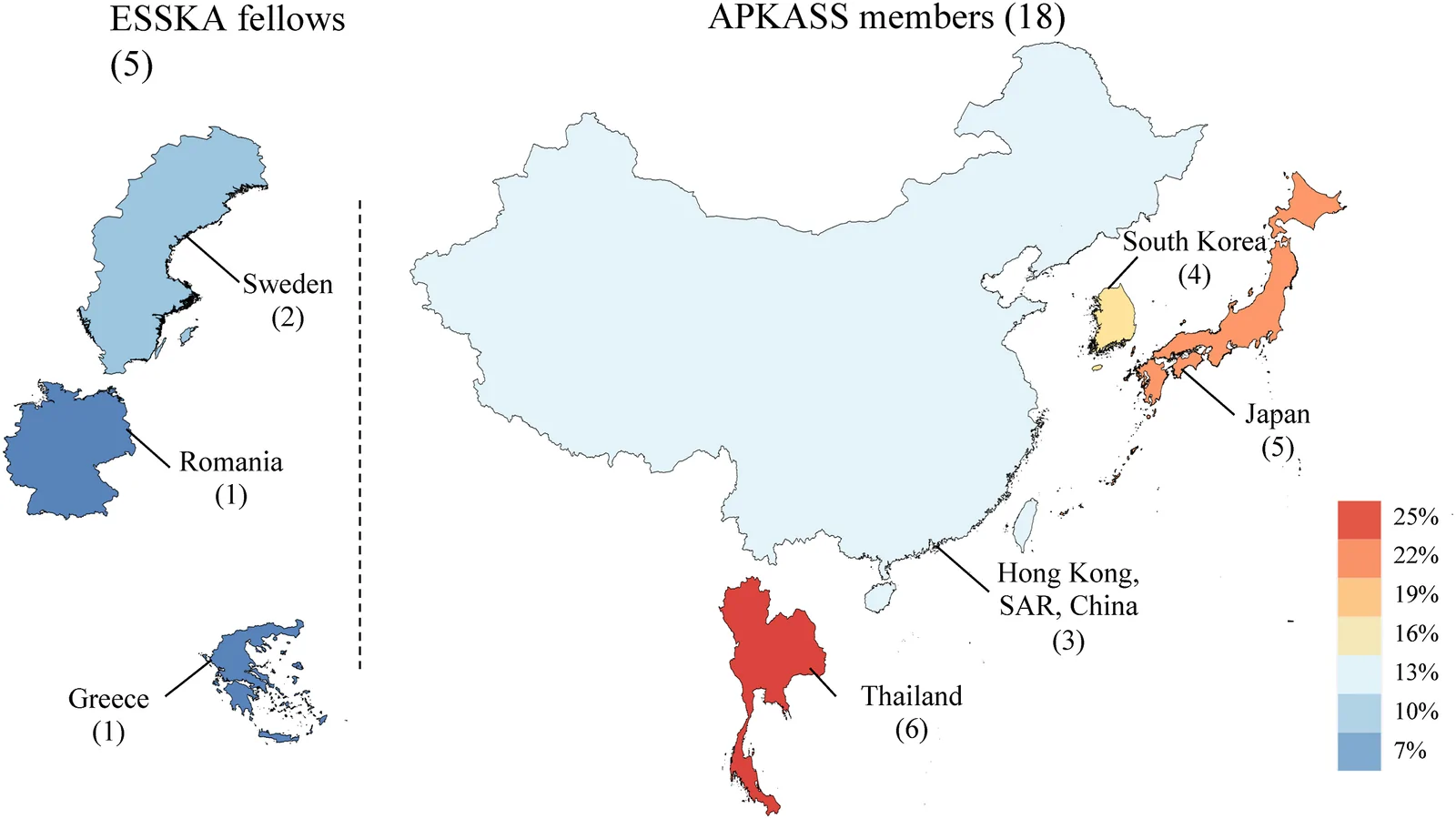
Distribution of participants by nationality and academic group.

This study was conducted by the authors through a single-round consensus meeting (24th Oct, 2024, Hong Kong, SAR) to investigate the APKASS experience and perspective about the most debatable topics in revision ACLR. 18 primary/revision ACL reconstruction surgeons from the APKASS group and 5 travelling fellows from ESSKA were invited (Fig. 1). The survey was completed by all participants, and the data were registered and stored in a database.

The colour scale shows the percentage of participants, and the actual number of participants is shown in parentheses.

The survey consisted of a questionnaire with 25 questions, with multiple choice. The questions dealt with the most controversial topics in revision ACLR. We break down the questions into 4 major aspects, including 1) Diagnostics and Preoperative Planning of Revision ACLR; 2) Indication for Revision ACLR; 3) Surgical techniques for Revision ACLR; 4) Rehabilitation and RTP of Revision ACLR. The questions are summarized in Table 1. We use percentage agreement as a way of evaluating the single-choice questions. The pre-defined threshold, 75%, must be met for a statement or item to be considered as achieving a consensus.^10^

**Table 1:** APKASS ACL revision Consensus questions.

Section	Question	Topic	Question Summary
Section 1: Diagnostics and Preoperative Planning			
	1	Failed ACL Definition	How is a failed ACL reconstruction defined? (Multiple criteria, including pivot shift, MRI findings, functional outcomes)
	2	Radiological Examination	Which radiological examinations should be performed for suspected failed ACL reconstruction? (X-ray, CT, MRI combinations)
	3	Tunnel Placement Assessment	How to assess tunnel placement and define tunnel misplacement? (X-ray, CT, MRI methods with specific measurements)
	4	Posterior Slope Measurement	How to measure the posterior slope of the medial tibial plateau? (Different anatomical axis methods)
	5	Femoral Tunnel Misplacement	Agreement on whether the misplaced femoral tunnel is the #1 cause of ACL graft failure
Section 2: Indication for Revision ACLR			
	6	Patient Activity Level	Does the indication for revision change with patient activity level? (Active vs sedentary lifestyle considerations)
	7	Patient Selection	Should ACL revision be performed in certain patient groups? (Age limits, osteoarthritis considerations)
Section 3: Surgical Techniques			
	8	Anatomic Approach	Is an anatomic approach possible in all primary reconstruction scenarios?
	9	Graft Choice - Primary HT	Most effective graft type for revision when the primary was the ipsilateral hamstring tendon
	10	Graft Choice - Primary BTB	Most effective graft type for revision when the primary was ipsilateral BTB
	11	Quadriceps Autograft	Agreement on soft tissue quadriceps autograft as a reliable choice for revision
	12	One-stage vs Two-stage	Percentage of revision surgeries performed with one-stage vs two-stage technique
	13	Two-stage Indications	Primary indication for choosing the two-stage technique (tunnel enlargement criteria)
	14	Double-bundle Failure	Surgical plan for failed double-bundle ACL reconstruction
	15	Femoral Tunnel Widening	Surgical plan for femoral tunnel widening ≥12 mm
	16	Lateral Extra-Articular Tenodesis	Necessity of LET in ACL revisions (based on pivot shift grade, patient age)
	17	High Tibial Osteotomy	Which ACL revision cases require combined HTO (compartment OA, varus, posterior slope)
	18	Femoral Tunnel Method	Preferred method for making femoral tunnel (trans-tibial, trans-portal, outside-in)
	19	Sagittal Malalignment	When is osteotomy needed to correct the posterior slope in revision surgery
	20	Over-The-Top Technique	Role of the Over-The-Top technique for revision ACL reconstruction
	21	Medial Knee Instability	How to deal with concomitant chronic medial knee instability (Grade 2)
	22	Meniscus Injury	How to deal with concomitant meniscus injury (repair vs staged procedures)
	23	Lateral Meniscus Root Tears	Preferred surgical technique for repairing LMPRTs in revision cases
Section 4: Rehabilitation and RTP			
	24	Rehabilitation Protocols	Should revision ACL patients have more conservative rehabilitation criteria than primary ACL patients
	25	Return to Play Rates	Return to play rates for ACL revision cases in the surgeon's series (<50% to >70%)

We further analyzed the current evidence with a systematic review to add further discussion in conjunction with the results of the expert consensus. The systematic review followed the updated PRISMA check-list.^10^ We used Pubmed, Embase, and the Scopus as search databases, with but not limited to the following queries: (Revisional ACL Surgery) or (Secondary ACL Reconstruction) or (Repeat ACL Surgery) or (ACL Revision Surgery) or (Revision Anterior Cruciate.

**Fig. 2:**
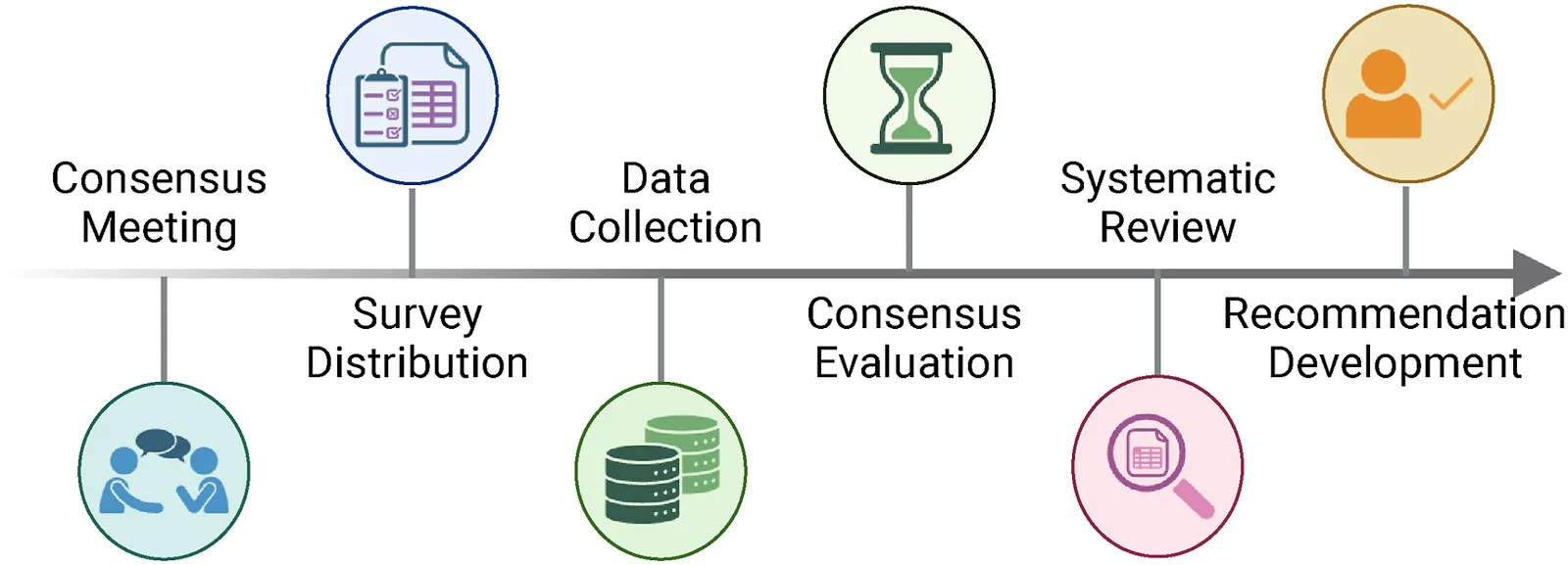
The overall consensus process.

Ligament Reconstruction) or (Re-operative ACL Reconstruction) (**supplement 2**). The authors subsequently summarize the literature and the responses to reach a consensus that resulted in practical recommendations for revision ACLR surgery. The overall process has been summarized in Fig. 2.

## 3 Discussion

The consensus was developed based on the personal experiences of 23 experts from the 8 member countries affiliated with APKASS & ESSKA. The topics on the most controversial issues related to revision ACLR were analyzed in light of the latest literature. The experts in this survey are all experienced surgeons, ensuring that the results reflect a broad spectrum of reliable and informed opinions on these complex topics. All items have been summarized in Table 2, Table 3, which present the consensus and non-consensus questions.

**Table 2:** Summary of consensus items (≥75% agreement).

Q#	Section	Survey Question	Consensus Statement (Agreement %)	Strength	Clinical Implication
Section 1*— Diagnostics and Preoperative Planning*					
Q1	Diagnostics	Definition of a failed ACLR (clinical/mechanical)	Positive pivot shift test + increased AP laxity (≥6 mm on KT-2000) defines graft failure	87.0%	Instrumented clinical examination remains the diagnostic cornerstone for graft failure.
Q1	Diagnostics	Definition of a failed ACLR (imaging)	MRI-defined graft retear is a reliable marker of failure	78.6%	MRI should be routinely performed in all suspected revision cases.
Q3	Diagnostics	Assessment of tunnel placement	CT scan is the preferred imaging modality for evaluating tunnel position and widening — 78.3%	78.3%	CT should be standard in preoperative planning for revision ACLR.
Q5	Diagnostics	Misplaced femoral tunnel as leading cause of graft failure	Misplaced femoral tunnel is the number-one cause of primary ACLR failure (Agree/Strongly Agree) — 82.1%	82.1%	Careful evaluation of prior femoral tunnel position is essential before revision.
Section 2 — ***Indications for Revision***					
Q6	Indications	Activity level & revision threshold (high-impact sports)	Patients engaged in high-impact/pivoting sports warrant a lower threshold for revision	91.3%	Activity demand is a principal determinant in revision decision-making.
Q6	Indications	Pre-injury activity and postoperative expectations	Pre-injury activity level strongly influences postoperative expectations and rehabilitation planning	87.0%	Patient counselling should be calibrated to pre-injury functional demands.
Section 3 — ***Surgical Techniques***					
Q21	Techniques — MCL	Concomitant chronic medial knee instability (Grade 2)	Revision ACLR combined with MCL reconstruction is recommended	91.3%	Multi-ligament reconstruction restores stability and protects the revised graft.
Q22	Techniques — Meniscus	Concomitant meniscus injury	All repairable meniscal tears should be repaired at the time of revision ACLR	100%	Meniscal preservation is a universally endorsed principle in the revision setting.
Q23	Techniques — LMPRT	Repair of lateral meniscus posterior root tears (LMPRTs)	One-stage independent transtibial pull-out repair is the preferred technique — 91.3%	91.3%	LMPRTs identified at revision should be addressed concurrently with independent transtibial fixation.
Section 4 — ***Rehabilitation and Return to Play***					
—	Rehabilitation/RTP	No items reached the ≥75% threshold	See Table 3 for the full distribution of responses (Q24, Q25).	—	Rehabilitation protocols and RTP thresholds remain heterogeneous; individualized approaches predominate.

Definition: Survey items for which a single response option reached or exceeded the pre-defined 75% agreement threshold among the 23 expert panellists (18 APKASS surgeons + 5 ESSKA travelling fellows).

Abbreviations: ACL, anterior cruciate ligament; ACLR, anterior cruciate ligament reconstruction; AP, anteroposterior; BTB, bone–patellar tendon–bone; CT, computed tomography; KT-2000, KT-2000 arthrometer; LMPRT, lateral meniscus posterior root tear; MCL, medial collateral ligament; MRI, magnetic resonance imaging; RTP, return to play.

Footnote: Consensus was defined a priori as ≥ 75% agreement among the 23 expert panellists. Items marked “combined” represent pooled “Agree” + “Strongly Agree” responses on Likert-scale questions.

**Table 3:** Summary of non-consensus items (<75% agreement).

Q#	Section	Survey Question	Most Selected Option (%)	Alternative Options (%)	Area for Future Research
Section 1*— Diagnostics and Preoperative Planning*					
Q1	Diagnostics	Definition of a failed ACLR — functional outcome (<90% LSI)	Supported — 65.2%	Not supported: 34.8%	Standardised cut-offs for functional failure.
Q1	Diagnostics	Definition of a failed ACLR — PROMs (KOOS <60, IKDC <70)	Supported — 30.4%	Not supported: 69.6%	PROMs alone insufficient; should be integrated with clinical/imaging findings.
Q2	Diagnostics	Required radiological work-up	AP/Lateral + WB X-ray + CT + MRI — 73.9%	Partial combinations: 26.1%	Near-consensus; standardised multi-modality imaging protocol recommended.
Q4	Diagnostics	Preferred method for measuring posterior tibial slope	CAA method — 39.1%	PAA: 34.8%; MAT: 21.7%; FSA: 4.4%	No universally accepted reference axis; biomechanical validation required.
Section 2***— Indications for Revision***					
Q6	Indications	Revision threshold for less active/sedentary patients	Non-operative management may suffice — 73.9%	Always consider surgery: 26.1%	Borderline; supports individualized activity-based decision-making.
Q7	Indications	Age cut-off/contraindications for revision	No strict age cut-off (perform regardless of age) — plurality	Age >60: 13.0%; Early OA excluded: 13.0%	Individualized rather than absolute contraindications.
Section 3***— Surgical Techniques***					
Q8	Techniques	Feasibility of anatomic revision approach in all scenarios	Feasible but not universally applicable — 47.8%	Universally feasible: 21.7%; Rarely feasible: 30.5%	Prior tunnels often preclude purely anatomic placement.
Q9	Techniques — Graft	Graft choice after primary ipsilateral hamstring failure	BTB (patellar tendon) autograft — 65.2%	QT: 13.0%; Allograft: 13.0%; Contralateral HT: 8.8%	BTB most favoured but not unanimous; selection remains individualized.
Q10	Techniques — Graft	Graft choice after primary ipsilateral BTB failure	Quadriceps tendon (QT) — plurality	HT; Allograft; Artificial ligament	Comparative data needed on QT vs. alternative grafts.
Q11	Techniques — Graft	Reliability of soft-tissue QT autograft in revision	Agree/Strongly Agree — 73.9% (combined)	Neutral/Disagree: 26.1%	Just below threshold; further comparative evidence required.
Q12	Techniques	One-stage vs. two-stage practice pattern	100% one-stage — 34.8%; 90% one-stage — 34.8%	80% one-stage: ∼17%; 70% one-stage: ∼13%	Strong trend toward one-stage; indications for two-stage remain heterogeneous.
Q13	Techniques	Primary indication for two-stage revision	Tunnel enlargement >18 mm (>100% initial diameter) — 47.8%	Tunnel >12 mm; infection-only; other: 52.2%	Clearer radiographic/volumetric criteria needed.
Q14	Techniques	Plan for failed double-bundle ACLR	Split between one-stage BTB, one-stage allograft, and two-stage if tunnel fusion	—	No consensus; requires case-based decision-making.
Q15	Techniques	Plan for femoral tunnel widening ≥12 mm	Mixed (one-stage interference screws/allograft/two-stage)	—	Threshold-based staging algorithm is needed.
Q16	Techniques — LET	Indication for Lateral Extra-Articular Tenodesis (LET)	Performed when pivot shift > Grade 2 — 65.2%	Always; Grade 3 only; Young high-risk: variable	Pivot shift is important but not the sole trigger for LET.
Q17	Techniques — HTO	Combined high tibial osteotomy (HTO) with revision ACLR	Never performed combined — 73.9%	AM OA; Varus >5°; Posterior slope >12°: 26.1%	HTO reserved for specific malalignment/slope indications.
Q18	Techniques	Preferred femoral tunnel drilling method	Case-by-case/Trans-portal (AM) — plurality	Transtibial; Outside-in	No single preferred technique in the revision setting.
Q19	Techniques	Osteotomy indication for sagittal malalignment (slope correction)	Posterior slope >12° + anterior tibial translation >5 mm — plurality	Slope >15°; >12° alone; combined criteria	Standardised slope threshold for slope-correcting osteotomy needed.
Q20	Techniques — OTT	Role of Over-The-Top (OTT) technique in revision ACLR	Prefer two-stage over OTT — 47.8%	Never performed OTT: 39.1%; Reliable option: 13.1%	OTT is an infrequently used salvage option need more evidence.
Section 4***— Rehabilitation and Return to Play***					
Q24	Rehabilitation	Rehabilitation criteria compared with primary ACLR	More conservative/patient-specific criteria — 52.2%	Parallels with primary: 30.4%; Psychological focus + similar progression: 17.4%	Individualized rehabilitation supported; no uniform protocol.
Q25	RTP	Reported RTP rate in revision ACLR cases	60–70% — 34.8%; >70% — 34.8%	<50%: 17.4%; 50–60%: 13.0%	Standardised RTP reporting needed; wide variability across cohorts.

Definition: Survey items for which no single response option reached the pre-defined 75% agreement threshold. For each item, the most-selected option (plurality response) is listed alongside alternative options to illustrate the distribution of expert opinion and highlight priorities for future research.

Abbreviations: ACLR, anterior cruciate ligament reconstruction; AM, anteromedial; AP, anteroposterior; BTB, bone–patellar tendon–bone; CAA, central anatomical axis; CT, computed tomography; FSA, fibular shaft axis; HT, hamstring tendon; HTO, high tibial osteotomy; IKDC, International Knee Documentation Committee; KOOS, Knee injury and Osteoarthritis Outcome Score; LET, lateral extra-articular tenodesis; LMPRT, lateral meniscus posterior root tear; LSI, limb symmetry index; MAT, mechanical axis of tibia; MCL, medial collateral ligament; MRI, magnetic resonance imaging; OA, osteoarthritis; OTT, over-the-top; PAA, proximal anatomical axis; PROM, patient-reported outcome measure; QT, quadriceps tendon; RTP, return to play; WB, weight-bearing.

Footnote: Consensus was defined a priori as ≥ 75% agreement among the 23 expert panellists. Items listed above did not meet this threshold and are presented to transparently report the diversity of expert opinion, identify areas of clinical equipoise, and highlight priorities for future research.

### 3.1 Section 1: Diagnostics and Preoperative Planning of Revision ACLR

For the “Diagnostics and Preoperative Planning of Revision ACL” part, the discussion focuses primarily on the definition of ACL failure, associated preoperative tests, and corresponding criteria. 87.0% of the experts identified a positive pivot shift test and increased anteroposterior laxity (≥6 mm on the KT-2000 arthrometer) as critical markers of failure, emphasizing their importance in assessing stability. Additionally, 78.6% recognized MRI-defined retear of the ACL graft as a valid criterion. Furthermore, 65.22% considered poor functional outcomes post-rehabilitation (measured by <90% Limb Symmetry Index) as indicative of failure, while only 30.4% supported using specific patient-reported outcomes (KOOS<60, IKDC<70) as standalone indicators. This variation in responses reflects the complexity of defining ACLR failure and suggests that establishing standardized criteria could improve clinical outcomes and patient management in clinical practice. Patient-reported outcomes (PROMs) like KOOS score (e.g KOOS-QoL <42) play a significant role in predicting graft failure.^11^ Meanwhile, by combining PROMs with specific clinical factors—such as graft choice and fixation methods—clinicians can better identify patients whose grafts are structurally intact but who still require revision surgery due to technical flaws like hardware impingement, improper tensioning, or non-anatomic placement.

There was a strong preference for comprehensive imaging regarding the radiological examinations necessary for assessing a known or suspected failed ACL reconstruction. 73.9% of experts support the combination of AP and lateral X-rays, weight-bearing (WB) X-rays, CT, and MRI as the most effective approach, consistent with the previous consensus.^6^ Some experts believe that CT is optional at this stage (3/23) and that long-leg WB x-ray can be replaced by scanogram (1/23), but this part failed to reach a general consensus.

There was a strong preference for using CT scans to assess tunnel placements in failed ACL reconstructions, supported by 78.3% of experts. CT is favoured for its superior visualization of bony structures and precise 3D measurements. While 17.4% support AP and lateral X-rays with specific measurement criteria (19-29% proximo-distal, 22-53% antero-posterior), and 26.1% opted for MRI, these methods are less preferred. Only 4.4% support X-ray assessment based on the Blumensaat line, due to its lower reliability.^12^ These results reflect a trend towards advanced imaging techniques for accurate tunnel evaluation, which is crucial given that tunnel malposition is a common cause of ACL reconstruction failure.

The findings reflect the continuous discussion in the field and show differing views on measuring the posterior slope (PTS) of the medial tibial plateau. The most supported method, with 39.1% of experts favouring it, involves calculating the angle between the central anatomical axis (CAA) and the line tangential to the anterior and posterior edges of the medial tibial plateau. This aligns with current literature emphasizing the importance of using a reliable anatomical reference axis for accurate measurements.^13^

The proximal anatomical axis (PAA) method, supported by 34.8% of experts, is also widely used and has shown good reliability in some studies. The mechanical axis of tibia (MAT) method, endorsed by 21.7%, is less common but still considered by some experts. The most widely accepted methods for measuring PTS are the PAA and CAA methods, and the literature reports that the difference between the two for the same patients may average up to 2°.^14^ This can lead to very different indications for revision surgery even if the surgeon follows the same PTS cut-off value (e.g., 12°). Corentin Pangaud et al. have used CT as well as multipoint localization to obtain a more accurate estimate of PTS.^15^

This variation in preferred methods highlights the complexity of measuring tibial slope accurately. Factors such as imaging modality (X-ray, CT, or MRI), visibility of anatomical landmarks, and the specific clinical context can influence method selection. The lack of a universally accepted standard emphasizes the need for further research to establish a consensus on the most reliable and reproducible technique for measuring medial tibial plateau slope, and also set the cut-off specifically for the Asian population, which is crucial for ACL procedures and biomechanical assessments. Epidemiology studies have reported that PTS may be higher in Asian populations than in Caucasian populations; however, this has not yet been reflected in the selection of cut-off values.^15^^,^^16^

More than 82.1% of experts agree that poor tunnel placement on the femoral side is the leading cause of ACL failure. While some experts believe reinjury may become the primary cause as surgical techniques advance, this has not yet become the prevailing opinion.

### 3.2 Section 2: Indication for Revision ACLR

Indications for revision ACLR vary significantly based on patient activity levels. A majority (91.3%) of experts agree that individuals involved in high-impact sports, such as soccer or basketball, have lower thresholds for revision surgery due to greater instability and functional demands. Conversely, 73.9% of experts believe that less active patients may not require revision, highlighting the role of activity level in determining surgical urgency. 87.0% of experts note that pre-injury activity levels influence post-revision expectations and rehabilitation goals, underscoring the importance of individualized clinical decision-making.^17^

In contrast, most experts (52.2%) believe that age does not affect revision ACLR decision-making as much as activity level does. While some experts agree with the concept, they still consider individuals older than 60 years of age (13.0%) and the established OA (13.0%) as relative contraindications to revision ACLR, despite retrospective studies showing a correlation between preoperative Kellgren-Lawrence grade and failure of revision ACLR.^18^

### 3.3 Section 3: surgical techniques for revision ACLR

The results of on the feasibility of an anatomic approach to revision ACLR reveal a mixed consensus among experts. A significant portion of experts, 47.8%, acknowledge that while an anatomic approach is feasible in many cases, it may not be universally applicable, indicating the necessity for case-specific considerations. In contrast, 21.7% strongly advocate for the universal applicability of these techniques across all primary reconstruction scenarios, while 26.1% remain neutral, reflecting uncertainty regarding their effectiveness. The complexities inherent in revision surgeries are often affected by factors such as previous tunnel placements and associated injuries. Anatomic reconstruction aims to restore the native ACL anatomy, which is crucial for enhancing knee stability and function; however, challenges like mal-positioned tunnels can significantly hinder its effectiveness. Thus, despite the preference for anatomic techniques due to their biomechanical advantages, a careful preoperative assessment and individualized planning are essential to ascertain their applicability on a case-by-case basis.

Regarding graft types for revision ACLR, experts clearly prefer bone-patellar tendon-bone (BTB), with 65.2% supporting its use. This aligns with existing literature that identifies BTB as the “gold standard” for ACL reconstruction due to its superior biomechanical properties and lower re-rupture rates compared to other graft types in primary cases.^19^ In a revision setting, a cohort study from the United States found that BTB was the preferred choice of surgeons, with approximately 57.1% choosing BTB as the graft for one-stage ACL revision cases.^20^ A systematic evaluation also suggested that BTB is the preferred procedure and showed superior results compared to tibialis anterior and Achilles allografts.^21^

13.0% of the experts favour quadriceps tendon (QT), while the allograft options receive support from 8.7% of respondents, as the least chosen. A Grassi et al. reported that autografts performed better than allografts, with decreased rates of complications, re-operations, and post-operative laxity. Although the results of autografts and allografts was comparable once irradiated allografts were eliminated,^22^ autologous grafts, represented by BTB, remain the dominant choice.

While hamstring tendon grafts are commonly used in primary ACLR, their efficacy in revision cases is debated; some studies suggest higher failure rates than BTB.^23^ Therefore, the decision regarding graft selection should consider factors such as the patient's activity level, previous graft performance, and individual anatomical considerations. These findings highlight the importance of selecting an appropriate graft type in revision ACL surgeries to optimize outcomes and minimize complications.

Moreover, there is growing recognition of the quadriceps tendon autograft as a reliable yet underappreciated option for ACLR and even in revision cases; 52.2% of experts agree on its potential benefits, with an additional 21.7% strongly agreeing. Research highlights that quadriceps tendon autografts can provide stability and functional outcomes comparable to those achieved with BTB grafts.^24^ Patients undergoing revision ACLR with quadriceps tendon autografts report satisfactory results,^25^ including quicker returns to sports and improved early patient-reported outcomes.^26^

Despite this positive outlook, 21.7% of respondents remain neutral regarding the widespread adoption of quadriceps tendon autografts, while only 4.4% disagree about their reliability. These findings suggest that while quadriceps tendon autografts may be underappreciated in clinical practice, they represent a viable alternative for revision ACLR. Increased awareness and further research could solidify their role as a preferred graft option for patients seeking effective outcomes with minimal complications following revision procedures. Overall, integrating these findings emphasizes the critical need for individualized approaches in surgical techniques and graft selection in revision ACLR to enhance patient outcomes effectively.

As for the one-stage/two-stages controversies, they reveal a strong preference for one-stage procedures in ACL reconstruction revision surgeries, with 34.8% of respondents indicating that they perform 100% of revisions in a one-stage manner and another 34.8% reporting 90% one-stage and 10% two-stage. This inclination towards one-stage revisions is attributed to their efficiency and reduced recovery times, minimizing the risks and complications associated with multiple surgeries. Varun Gopinatth et al. did a meta-analysis that included 355 patients of two-stage ACLR, found that the primary indications were tunnel widening (>14 mm) and mal-positioning, and that good outcomes could be achieved despite high variability in technique.^27^ In the present consensus, 47.8% of experts advocate for two-stage revisions in cases of significant tunnel enlargement, particularly when tunnels exceed 14 mm, which complicates graft placement and integration. Notably, 21.7% of the experts only did the two-stage revision in infection cases. With the use of bony grafts such as BTB and bony quadriceps grafts to manage borderline cases of tunnel enlargement (12-14 mm), one-stage reconstruction would obviously be a more acceptable surgical option for the patient.

The addition of Lateral Extra-articular Tenodesis (LET) to ACLR is increasingly recognized for its benefits in improving knee stability, particularly in patients at higher risk for reinjury.^28^^,^^29^ In the present study, most experts (65.2%) agree that the pivot shift test is the primary indicator for LET.^30^43.5% of the experts believe that cases with grade II or above pivot shift require additional LET procedure. Some additional considerations, such as joint laxity, high activity level, PTS> 12°, and un-reparable meniscus, have been proposed to be the indications. Anterolateral ligament reconstruction (ALLR), compared with lateral extra-articular tenodesis (LET), is not commonly used in multiple centers and therefore has not been discussed separately. Current biomechanical evidence suggests that both procedures have similar effects in reducing rotational and pivot-shift instability.^31^ Overall, surgeons shall feel that there is a role for both LET and ALLR procedures.

Previous studies have demonstrated that in individuals with an ACL injury, greater knee rotatory laxity is associated with damage to the anterior lateral complex, medial/lateral meniscus.^32^ LET may offer additional value in restoring knee stability for these patients. Future research should focus on further analyzing the optimal indications for LET in revision ACLR through prospective studies.

High tibial osteotomy (HTO) combined with ACLR is indicated primarily for young, active patients with medial tibiofemoral OA/varus deformity and symptomatic ACL deficiency.^33^^,^^34^ 73.9% of experts indicated they have never performed ACL revision with HTO. Among those who consider the combination, 47.8% support it primarily for cases of anterior medial compartment OA accompanied by varus deformity greater than 5°, while 17.4% cite an increased posterior tibial slope greater than 12° as a relevant indication.

HTO can effectively correct malalignment and address the increased PTS, which has been shown to predispose patients to graft failure. Mabrouk A et al. reported that HTO (Anterior closing wedge) combined with ACLR is effective in restoring knee stability and improving function with an acceptable rate of specific complications.^35^ Interestingly, this study recruited revision patients with PTS >12° who participated in impact sports and high-impact sports. In summary, combined HTO and revision ACLR should be considered in select cases with significant alignment issues.

Similarly, APAKSS experts do not support the use of Over-The-Top (OTT) technique in revision cases. 47.8% of the experts preferred to solve complex femoral tunnel problems with two-stage surgery, whereas 39.1% had never done OTT. The OTT approach does not replicate the natural anatomy of the ACL, which is a significant drawback.^36^ Only 21.7% of the experts considered it to be a viable option in complex cases.

A consensus was reached regarding the repair of concomitant injuries to other parts of the joint, such as the meniscus and medial compartment. 100% of the experts supported the idea that all repairable menisci should be repaired, which was consistent with the previous ESSKA consensus.^9^ For special cases, such as lateral meniscus posterior root tears (LMPRTs), 91.30% of the experts supported using one-stage and independent transtibial pullout procedures for fixation. A tear of the LMPRTs is found with significantly greater frequency in revision ACLR (20.5% vs 5.6%) compared to primary reconstructions,^37^ often due to the recurrent instability and trauma the knee has endured.^38^ While there is a general agreement that repairing these tears is crucial to restoring the meniscus's function as a secondary stabilizer and preventing early-onset arthritis, the “how” and “when” are subjects of considerable debate.

For chronic medial knee instability, the ACLR + MCL reconstruction approach was also supported by 91.3% of experts, and recognized as the gold standard.^39^ The preference is further substantiated by evidence showing that MCL reconstruction leads to lower failure rates in patients undergoing combined revision ACLR and chronic medial instability, as compared with MCL repair.^38^

### 3.4 Section 4: rehabilitation and RTP for revision ACLR

The results regarding rehabilitation protocols for revision ACLR patients indicate a significant preference for more conservative criteria compared to primary ACL reconstruction patients, with 52.2% of respondents supporting the idea that progression criteria should be more conservative than primary case, emphasizing patient-specific criteria over strict timelines. This approach acknowledges the complexities associated with revision surgeries, where prior injuries and surgeries can lead to altered knee mechanics and increased risk of reinjury.

While 30.4% of experts note that psychological factors, such as fear of reinjury, play a crucial role in rehabilitation, they believe that the overall progression criteria remain similar to those for primary ACL reconstruction. However, it is essential to recognize that revision ACLR patients often face unique challenges, including elevated levels of anxiety and concerns about returning to sport, which can impact their recovery and adherence to rehabilitation protocols.

Current literature supports the notion that rehabilitation for revision ACLR should be tailored to address these psychological factors and the patient's specific history. A conservative approach allows for adequate healing time and focuses on building strength and neuromuscular control before progressing to more demanding activities. Studies have shown that a longer rehabilitation timeline can lead to better long-term outcomes, including reduced reinjury rates and improved functional performance. Overall, these findings emphasize the importance of individualized rehabilitation strategies in managing revision ACLR cases, ensuring that both physical and psychological aspects are adequately addressed to optimize recovery and return to sport.

The results regarding return-to-play (RTP) rates for revision ACLR cases indicate a diverse range of outcomes, with 34.8% of respondents reporting RTP rates of 60% to 70%, and another 34.8% indicating rates greater than 70%. In contrast, 17.4% reported RTP rates of less than 50%, while 13.0% noted rates between 50% and 60%. These findings reflect the variability in RTP outcomes following A revision ACLR, which can be influenced by numerous factors, including the severity of the initial injury, the type of graft used, and the presence of concomitant injuries.

Current literature supports these findings, showing that the RTP rate for athletes following revision ACL reconstruction can range significantly, with studies reporting rates from 56% to 100% for returning to any level of sport, and between 13% and 69% for returning to pre-injury levels. Factors such as psychological readiness, age, and the specific demands of the sport play critical roles in determining these outcomes. For instance, athletes often face challenges related to fear of reinjury and may require additional psychological support during rehabilitation.

This study also has several limitations. First, we used a single-round survey to establish consensus rather than a multi-round Delphi process; therefore, relatively few items reached the ≥75% consensus threshold in single round. In addition, although the 25 questions were designed to cover various aspects of revision ACLR, they were not fully comprehensive. Future studies will attempt to address this limitation and may expand the inclusion of APKASS representatives in order to achieve recommendations with a higher level of evidence.

Overall, while a substantial proportion of athletes can successfully return to sport after revision ACLR, many do not achieve their previous level of performance. This marks the importance of tailored rehabilitation protocols that address both physical recovery and psychological factors to optimize RTP outcomes in this population.

## 4 Conclusion

This study highlights the consensus among surgeons from APKASS and ESSKA regarding key aspects of revision ACL reconstruction, emphasizing the complexity of managing these cases. The findings highlight the importance of standardized diagnostic criteria, comprehensive imaging techniques, and individualized surgical planning to address factors such as tunnel placement, tibial slope, and patient-specific activity levels. Despite varying preferences for graft types and surgical techniques, BTB and quadriceps tendon autografts emerged as reliable options, with one-stage procedures favoured in most cases unless significant tunnel enlargement necessitates a two-stage approach.

Rehabilitation strategies required a more conservative, patient-specific approach, addressing both physical and psychological aspects to optimize outcomes. While return-to-play rates vary significantly, tailored protocols and psychological support are critical for achieving functional recovery and minimizing reinjury risks.

Overall, this consensus highlights the need for individualized, evidence-based strategies in revision ACL reconstruction to improve patient outcomes, ensure knee stability, and facilitate successful return to sport. Future research should focus on refining diagnostic standards, exploring optimal surgical techniques, and developing targeted rehabilitation protocols to further advance the field.

## Availability of data and materials

The data and materials are available from the corresponding author upon reasonable request.
