# Supplementary material for: APKASS 2024 consensus statement on anterior cruciate ligament reconstruction, Part II: Management of anterior cruciate ligament revision in adults
Source: Asia Pac J Sports Med Arthrosc Rehabil Technol. 2026 Aug 13;45:74–81. doi: 10.1016/j.asmart.2026.06.001 (PMC13495588; doi:10.1016/j.asmart.2026.06.001)
Supplement: Supplementary file 1 [file mmc1.docx]

**Consensus on Anterior Cruciate Ligament Reconstruction, Part II: Management of Anterior Cruciate Ligament Revision in Adults**

***Section 1: Diagnostics and Preoperative Planning of Revision ACL***

1. ***How is a failed ACL reconstruction (ACLR) defined?*** (***Check all that apply)***

*□ A.* *Pivot shift test (+),* *increased anteroposterior laxity (≥6 mm) on KT-2000 arthrometer*

*□ B. MRI defined retear of the ACL graft*

*□ C. Poor function directly related to ACL reconstruction* *after rehab (< 90% LSI)*

*□ D. Poor function directly related to ACL reconstruction after rehab (KOOS<60, IKDC<70), can’t return to sports.*

*□ E.* *Others ___________ please specify*

2. ***Which radiological examination should be performed/documented in the setting of a known or suspected failed ACL reconstruction?***

*□ A. A**P and* *Lateral X-ray + WB X-ray*

*□ B. AP and Lateral X-ray + WB X-ray + stress X-ray*

*□ C. AP and Lateral X-ray + WB X-ray + CT*

*□ D. AP and Lateral X-ray + WB X-ray + CT + MRI*

*□ E. Others ___________ please specify*

3. ***How can the assessment of tunnel placement be performed, and what is the definition of tunnel misplacement in the setting of a known or suspected failed ACL reconstruction?*** (***Check all that apply)***

*□ A. AP and LL X-ray**, the* *centre is outside 19–29% of the proximo-distal dimension and 22–53% of the antero-posterior dimension*

*□ B. AP and LL X-ray, when the tunnel is anterior to the Blumensaat line in full knee extension.*

*□ C. CT scan*

*□ D. MRI scan*

4. ***How to measure the posterior slope of the medial tibia plateau?***

*□ A. Calculating the angle between proximal anatomical axis (PAA) and the line tangential to the anterior and posterior edges of the medial tibial plateau.*

*□ B. Calculating the angle between mechanical axis of tibia* *(MAT) and the line tangential to the anterior and posterior edges of the medial tibial plateau.*

*□ C. Calculating the angle between central anatomical axis (CAA) and the line tangential to the anterior and posterior edges of the medial tibial plateau.*

*□ D. Calculating the angle between fibular shaft axis (FSA) and the line tangential to the anterior and posterior edges of the medial tibial plateau.*

5. ***The misplaced femoral tunnel is the number one cause of anterior cruciate ligament reconstruction graft failure?***

*□ A. Strongly Agree*

*□ B. Agree*

*□ C. Neutral*

*□ D. Disagree*

*□ . __________________________________________ please specify reason*

***Section 2: Indication for Revision ACL***

6. ***Does the indication for revision ACL reconstruction change with the activity of the patient? (Check all that apply)***

*□ A. Patients who lead a less active lifestyle or those who do not engage in cutting or pivoting sports may not require revision surgery,*

*□ B. Patients engaged in high-impact sports (e.g., soccer, basketball) often have different thresholds for revision surgery compared to those participating in low-impact activities.*

*□ C. Patients who are more active before their injury may have different expectations and rehabilitation needs post-revision.*

*□ D. Others ___________ please specify*

7. ***Should ACL revision be performed in a certain group of patients?***

*□ A. ACL revision surgery shall not be performed in patient over 60*

*□ B. ACL revision surgery shall not be performed in patient over 55,*

*□ C. ACL Revision surgery should be performed regardless of the patient's age*

*□ D. Patients with early signs of OA do not benefit from revision surgery,*

*□ E. Others ___________ please specify*

***Section 3: Surgical techniques for Revision ACL***

8. ***Anatomic approach to a revision ACL surgery is possible in all the primary reconstructions existent scenarios?***

*□ A. Strongly Agree*

*□ B. Agree*

*□ C. Neutral*

*□ D. Disagree*

*□ . __________________________________________ please specify reason*

9. ***Which graft type is generally considered most effective for revision ACL surgery (Primary: Ipsilateral*** ***hamstring tendon)?***

*□ A. Quadriceps Tendon (QT)*

*□ B. Contralateral hamstring tendon* *(HT)*

*□ C. Patellar tendon (PT)*

*□ D. Allograft*

10. ***Which graft type is generally considered most effective for revision ACL surgery (Primary: Ipsilateral BTB)?***

*□ A. Quadriceps Tendon (QT)*

*□ B. Hamstring tendon (HT)*

*□ C. Artificial ligament (AL)*

*□ D. Allograft*

11. ***Soft Tissue Quadriceps Autograft is a Reliable and underappreciated graft choice for revision ACL surgery?***

*□ A. Strongly Agree*

*□ B. Agree*

*□ C. Neutral*

*□ D. Disagree*

*□ . __________________________________________ please specify reason*

12. ***What is the percentage of the ACL reconstruction revision surgeries do you perform with the one-stage/two-stage technique?***

*□ A. 100% one-stage; 0% two-stage*

*□ B. 90% one-stage; 10% two-stage*

*□ C. 80% one-stage; 20% two-stage*

*□ D. 70% one-stage; 30% two-stage*

13. ***What is the primary indication for your two-stage cases?***

*□ A. Tunnel enlargement greater than 12 mm;*

*□ B. Tunnel enlargement greater than 14mm, greater than 100% of the initial tunnel diameter*

*□ C. I only did two-stage in infection case*

*□ D. Others ___________ please specify*

14. ***What is the surgical plan suitable for a failed double-bundle ACL Reconstruction case?***

*□ A.* *One-stage revision with the bone block (BTB* *autograft)*

*□ B. One-stage revision with the allograft*

*□ C. If bone tunnel fusion is present, two-stage revision*

*□ D. Others ___________ please specify*

15. ***What is the surgical plan suitable for a femoral tunnel widening to 12mm or more?***

*□ A. One-stage revision with the interference screws*

*□ B. One-stage revision with the allograft*

*□ C. Two-stage revision*

*□ D. Others ___________ please specify*

16. ***What is the current stance on the necessity of Lateral Extra-Articular Tenodesis (LET) in ACL revisions?*** (***Check all that apply)***

*□ A. I always perform irrespective the pivot shift test grade.*

*□ B. I perform only in cases with pivot shift test > grade 2.*

*□ C. I perform only in cases with grade 3 pivot shift test.*

*□ D. I perform in young patients with a risk of reinjury.*

17. ***Which ACL revision cases require combined high tibial osteotomies (HTO)***

*□ A. AM compartment OA only.*

*□ B. AM compartment OA, varus > 5°.*

*□ C. Posterior tibial slope > 12°*

*□ D. I never did the ACL revision with HTO*

*□ E*. Others ___________ please specify

18. ***What is the preferred method to make the femoral tunnel?***

*□ A. Trans-tibia*

*□ B. Trans-portal (antero-medial)*

*□ C. Outside-in*

*□ D. Decision case by case*

*□ E.* *Others ___________ please specify*

19. ***When is osteotomy needed to correct the sagittal malalignment (slope) in ACL revision surgery***

*□ A. Posterior slope >15°,*

*□ B. Posterior slope >12°,*

*□ C. Posterior slope >12°, & anterior tibia translation > 5mm*

*□ D. Posterior slope >12°, & anterior tibia translation > 5mm, without hyper-extension*

20. ***Is there a role of Over-The-Top*** ***Technique for Revision ACL Reconstruction?***

*□ A. The Over-The-Top Technique represents a reliable option for revision ACL reconstruction, particularly in complex cases involving tunnel mal-positioning or rotatory instability.*

*□ B. I prefer the two-stage surgery over the OTT Technique.*

*□ C. I never use the over-the-top technique and have no comment.*

*□ D. Others ___________**please specify*

21. ***How to deal with the revision ACL reconstruction with concomitant chronic medial knee instability (Grade 2)?***

*□ A. ACL revision only.*

*□ B. ACL revision + MCL repair*

*□ C. ACL revision + MCL reconstruction*

*□ D. Others ___________please specify*

22. ***How to deal with the revision ACL reconstruction with concomitant meniscus injury?*** (***Check all that apply)***

*□ A. All case, combined ACL revision + meniscus repair if repairable*

*□ B. For locked knee due to an acute injury, staged procedures,*

*□ C. Small asymptomatic meniscal lesions can be left untreated*

*□ D. Others ___________please specify*

23. ***For revision ACL reconstruction, what is the preferred surgical technique for repairing lateral meniscus posterior root tears (LMPRTs)?***

*□ A. One-stage, independent transtibial pullout repair*

*□ B. Shared tibia bone tunnel technique*

*□ C. Two-stage, independent transtibial pullout repair*

*□ D. Others ___________please specify*

***Section 4: Rehabilitation and RTP for Revision ACL***

24. ***For ACL revision patient, they shall have a more conservative*** ***criteria of rehabilitation protocols compared with the primary ACL reconstruction patient.***

*□ A. Rehabilitation protocols for ACL revision surgery often draw parallels with those for primary ACL reconstruction.*

*□ B. Given the history of prior injury, there is often a greater emphasis on addressing psychological factors such as fear of re-injury, but the progression criteria are similar.*

*□ C. The progression criteria are shall be more conservative, emphasizing patient-specific criteria over strict timelines*

*□ D. Others ___________please specify*

25. ***The Return to play (RTP) rates for the ACL revision case in your series***

*□ A. <50%*

*□ B. 50%~60%*

*□ C. 60%~70%*

*□ D. >70%*

*E. Others ___________please specify*
