# Supplementary material for: APKASS 2024 consensus statement on anterior cruciate ligament reconstruction, Part II: Management of anterior cruciate ligament revision in adults
Source: Asia Pac J Sports Med Arthrosc Rehabil Technol. 2026 Aug 13;45:74–81. doi: 10.1016/j.asmart.2026.06.001 (PMC13495588; doi:10.1016/j.asmart.2026.06.001)
Supplement: Supplementary file 2 [file mmc2.docx]

**Supplement 2.1**

Search strategy:
**Pubmed:** ("Anterior Cruciate Ligament"[Mesh] OR "Anterior Cruciate Ligament Reconstruction"[Mesh] OR "Anterior Cruciate Ligament"[tiab] OR "Anterior Cruciate Ligaments"[tiab] OR ACL[tiab] OR ACLR[tiab]) AND (reconstruction*[tiab] OR repair*[tiab] OR surgery[tiab] OR operative[tiab] OR graft*[tiab]) AND ("Reoperation"[Mesh] OR "Treatment Failure"[Mesh] OR revision*[tiab] OR reoperation*[tiab] OR "re-operation*"[tiab] OR fail*[tiab] OR rerupture*[tiab] OR "re-rupture*"[tiab] OR recurrent[tiab])

N=6,216

**Embase:** (exp anterior cruciate ligament/ OR exp anterior cruciate ligament reconstruction/ OR (anterior cruciate ligament* OR ACL OR ACLR).ti,ab.) AND (reconstruction* OR repair* OR surgery OR operative OR graft*).ti,ab. AND (exp reoperation/ OR exp treatment failure/ OR exp graft failure/ OR (revision* OR reoperation* OR re-operation* OR fail* OR rerupture* OR re-rupture* OR recurrent).ti,ab.)

N=1,910

**Scopus:** TITLE-ABS-KEY ( ( "Anterior Cruciate Ligament*" OR acl OR aclr ) AND ( reconstruction* OR repair* OR surgery OR operative OR graft* ) AND ( revision* OR reoperation* OR "re-operation*" OR fail* OR rerupture* OR "re-rupture*" OR recurrent ) )

N= 8,302

**Supplement 2.2**

**Identification of studies via databases and registers**

Records removed *before screening*:

Duplicate records removed (n = 6,692)

Records identified from*:

Databases (n = 9,736)

Pubmed = 6,216

Scopus = 8,302

Embase = 1,910

**Identification**

Records excluded**

(n = 1,612) News, perspective, correspondence, corrigendum

Records screened

(n = 8,124)

Reports not retrieved

(n = 4,400) Pre-clinical study; did not involve the topic discussed in the consensus.

Reports sought for retrieval

(n = 6,512)

**Screening**

Reports assessed for eligibility

(n = 2,112)

Reports excluded:

(n = 1,344) Without data to support the present consensus

Studies included in review

(n = 768)

**Included**

**Supplementary 2.2**. PRISMA flow diagram of included studies
